# The seahorse (*Hippocampus comes* L.) extract ameliorates sperm qualities, testosterone level, and serum biochemistry in rats induced by depo medroxyprogesterone acetate

**DOI:** 10.5455/javar.2023.j661

**Published:** 2023-03-31

**Authors:** Trisnawati Mundijo, Franciscus Dhyanagiri Suyatna, Agung Eru Wibowo, Silvia Werdhy Lestari, Yusra Yusra, Yurnadi Hanafi Midoen

**Affiliations:** 1 Doctoral Programme Biomedical Science, Faculty of Medicine, Universitas Indonesia, Jakarta, Indonesia; 2 Department of Medical Biology, Faculty of Medicine, Universitas Muhammadiyah Palembang, Palembang, Indonesia; 3 Department of Pharmacology and Therapeutics, Faculty of Medicine, Universitas Indonesia, Jakarta, Indonesia; 4 Research Centre for Pharmaceutical Ingredient and Traditional Medicine, National Research and Innovation Agency, Tangerang, Indonesia; 5 Department of Medical Biology, Faculty of Medicine, Universitas Indonesia, Jakarta, Indonesia; 6 Department of Clinical Pathology, Faculty of Medicine, Universitas Indonesia, Jakarta, Indonesia

**Keywords:** DMPA, seahorse, serum biochemistry, spermatozoa, testosterone

## Abstract

**Objective:**

The percentage of infertility cases in this world is about 50%. Seahorses (*Hippocampus* spp.) are widely used in traditional medicine. Several studies suggest that seahorses have ethnopharmacological characteristics, such as fertility, antioxidants, and antifatigue. The purpose of this study was to determine whether seahorse extract (SE) (*Hippocampus comes* L.) has an effect on fertility and serum biochemistry in rats induced by depo medroxyprogesterone acetate (DMPA).

**Materials and Methods:**

All animals were induced with 1.25 mg/kg BW of DMPA. Animals were grouped into five groups: namely aquadest, 1% CMC, and SE doses of 150, 225, and 300 mg/kg BW. The rats were gavage every morning from week 7 until 18. At the end of our study, semen from the vas deferens and blood from the heart were analyzed. We analyzed with a one-way analysis of variance and Bonferroni’s post hoc tests (α 95%).

**Results:**

The concentration of spermatozoa had a significant difference in dose of 150 mg/kg BW compared to other groups (*p = *0.04). In contrast, the motility (*p *= 0.012) and viability of spermatozoa (*p = *0.007) were highly significant differences (*p < *0.05 and *p < *0.01) at 300 mg/kg BW. Testosterone levels were not significantly (*p* = 0.162; *p* > 0.05), but tended to increase at 300 mg/kg BW (11.01%). Nevertheless, serum biochemistry was insignificant (*p > *0.05) in all groups.

**Conclusion:**

SE (*Hippocampus comes* L.) ameliorates fertility and serum biochemistry in rats induced by DMPA.

## Introduction

To date, infertility is a health problem in 187 million couples worldwide [1]. The World Health Organization (WHO) defines infertility as the inability of a couple to conceive pregnant after at least 1 year of regular and unprotected sexual intercourse [2]. Worldwide, this condition occurs in almost 155 couples, 50% of which are male factor [3–5]. Male infertility could affect spermatogenesis and testosterone levels, which decreases semen quality and has medical implication for social and emotional stigma [6,7].

In this decade, traditional medicine therapies for infertility have increased. WHO encourages the rational use of herbal medicines, but scientific data on their use is still limited. Therefore, it still needs to be explored which products affect fertility, including marine natural products (MNPs), there is a seahorse [8,9].

Seahorse (genus *Hippocampus *sp*.*) is a teleost fish, family Syngnathidae which is useful for curing infertility, has a hormone-like effect, and is antioxidant, anti-inflammatory, antifatigue, and a trade commodity [10–12]. Seahorses have steroids, protein, trace elements, amino acids, cholesterol, and fatty acids [13,14]. Recently, many studies have shown the effects of seahorses. Xu et al. [15] stated that seahorse extract (SE) is a candidate marine drug for Benigna Prostate Hyperplasia without side effects. Park et al. [16] reported an increase in serum testosterone levels in mice with seahorse. Another study reported the hydrolysate protein of seahorse (*Hippocampus kuda*) amelioration effects on diabetic rats with reproductive dysfunction [17]. The benefit of seahorses must have safety for human beings, one of which is through the serum biochemistry evaluation. There are no reports of serum biochemistry, especially *Hippocampus comes *L. in Indonesia, so further exploration is still needed.

DMPA affects the endocrine system, one of which inhibits the gonadotropic pituitary from producing the hormone testosterone [18]. It is suspected that biocompounds in seahorse can increase the hormone testosterone, nevertheless, studies are needed to find out this, which is why this study was carried out.

## Materials and Methods

Our study from April until September 2021 is described.

### Ethical approval 

The ethics from the ethics committee of the Faculty of Medicine, Universitas Indonesia (KET-101/UN2.F1/ETIK/PPM.00.02/2021).

### Animals and treatment

The SE in this study was derived from our collection of materials. Sprague-Dawley male rats (SD; 200–250 gm; 8 weeks old) from the Agency for Drug and Food Control in Indonesia, and acclimatized for 1 week, treated, and maintained for 18 weeks in the Faculty of Medicine, Universitas Indonesia, at Animal Research Facilities-Indonesian Medical Education and Research Institute.

All animals were placed at room temperature 25°C, 12 h (light-dark cycle), with food and water access. After acclimatization, the rats were randomly divided into 5 groups (at 6 animals) aquadest (G1), CMC 1% (G2), SE dose 150 mg/kg BW (G3), 225 mg/kg BW (G4), and 300 mg/kg BW (G5). All animals were injected 1.25 mg/kg BW DMPA intramuscularly, alternately into the right or left thigh, at week 0 and 12. SE was administrated for treatment from week 7 to 18. In our last study, we euthanized all animals with ketamine Ket-A-100 and Xylazine Xyla Holland. 

### Assessment of testosterone levels and serum biochemistry

After euthanasia, 2.5 ml of blood from the heart was taken directly with taken with a syringe and transferred into a vacuum tube without Ethylene Diamine Tetra-acetic Acid. Blood in vacuum tubes was centrifuged at 3,500 rpm for 10 min to obtain blood serum, and analyzed by Enzyme-Linked Immunosorbent Assay (ELISA) with immulite molecular devices, *V*max kinetic microplate reader (USA), and ELISA Kit Rat Testosterone (T) Cusabio Lot.C2261102250 following kit instructions. Parameter Serum biochemical parameters were analyzed using flow cytometry for high-density lipoprotein (HDL), low-density lipoprotein (LDL), aspartate aminotransferase (AST), alanine amino transferase (ALT), cholesterol, and triglyceride parameters with the chemistry analyzer MNCHIP Pointcare M4kit (China). 

### Assessment of spermatozoa parameter

Immediately after the blood was drawn, semen from the right and left vas deferens was squeezed and diluted on a glass deck with 1% NaCl solution as a suspension. Suspended sperm were counted and assessed by WHO methods [19]. The concentration, motility, and viability of spermatozoa were observed using a Neubauer’s hemocytometer under a light microscope at 400× magnification. Sperm concentration was determined from a suspension of sperm diluted with George’s solution, calculated by the formula, and expressed in million/ml. The motility of spermatozoa was classified as motile or immotile and expressed as a percentage. Spermatozoa viability was defined by preparing sperm suspension and 3% eosin-Y solution with the percentage of live spermatozoa (colorless spermatozoa) in 100 sperm.

### Statistical analysis

Data was collected and tabulated to analyze using the IBM Statistical program for social science statistics for windows ver. 20 and mean SD with *p < *0.05.

## Results

### Effects of SE on rat’s fertility

The group that was given SE had a higher concentration of spermatozoa than the group that was not given SE, but it decreased with increasing doses of the extract (Table 1). G3 showed the highest concentration (52.60 × 106/ml), significantly different from G5 (30.90 × 10^6^/ml). (*p *< 0.05). In contrast to the concentration parameters, the highest motility was in the G5 (62.00%) compared to other groups and significantly increased (*p *< 0.05) compared to the G2 of about 48.90% and G3 of about 48.30%.

In addition, the parameters of spermatozoa viability gave the same results as sperm motility. We significantly found the highest spermatozoa viability in group G5 (64.10%) when compared to other groups (*p* < 0.05) compared to G2 (51.80%) and G3 (53.10%).

**Figure 1.**
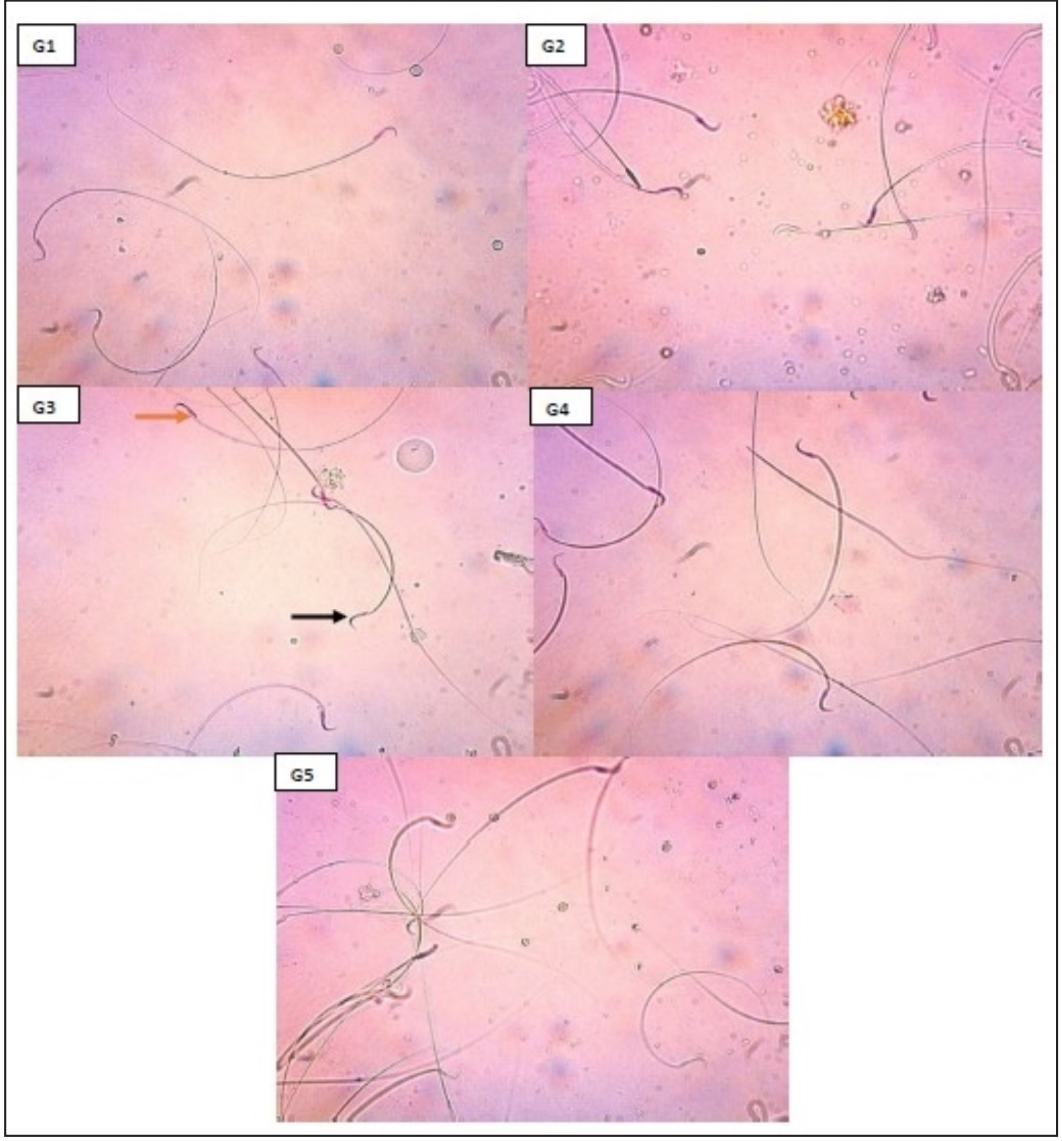
Viability of spermatozoa. Magnifying microscope 400×. G1: Aquadest, G2: CMC 1%, G3: SE dose of 150 mg/kg BW, G4: SE dose of 225 mg/kg BW, G5: SE dose of 300 mg/kg BW. → : not-viable spermatozoa, → : viable spermatozoa.

The results of spermatozoa viability as shown in Figure 1 showed that spermatozoa viability was characterized by stained and unstained spermatozoa heads. Stained spermatozoa heads indicate that the spermatozoa have died. This causes the cell membrane to be penetrated by the dye, while the unstained spermatozoa heads indicate that the spermatozoa are still alive with the cell membrane intact so that the dye cannot be penetrated. 

**Table 1.** Concentration, motility, and viability of spermatozoa, and testosterone levels in rats.

Parameter	G1	G2	G3	G4	G5	*p-*value
Spermatozoa concentration (10^6^/ml) Mean ± SD	39.90 ± 9.80	36.30 ± 11.38	52.60 ± 10.04	34.40 ± 12.68	30.90 ± 8.94	0.040*
Spermatozoa motility (%) Mean ± SD	55.60 ± 9.37	48.90 ± 5.44	48.30 ± 4.08	57.20 ± 6.08	62.00 ± 5.14	0.012*
Spermatozoa viability (%) Mean ± SD	61.10 ± 6.01	51.80 ± 5.66	53.10 ± 4.17	59.20 ± 6.07	64.10 ± 4.35	0.007**
Testosterone level (ng/ml) Mean ± SD	8.32 ± 5.36	6.85 ± 4.95	11.01 ± 7.03	6.75 ± 2.65	3.26 ± 2.19	0.162

Data with a mean ± SD. G1: Aquadest, G2: CMC 1%, G3: SE dose 150 mg/kg BW, G4: SE dose 225 mg/kg BW, G5: SE dose 300 mg/kg BW. *Significant difference *p < *0.05, **Highly significant difference *p < *0.01.

According to Table 1, various doses of SE had no significant effect influence (*p = *0.162; >0.05) on the testosterone levels in rats compared to all groups without SE administration. However, G3 tended to have the highest testosterone levels (11.01 ng/ml) compared to other groups. 

### Serum biochemistry level

Serum biochemistry level in our study included HDL, LDL, AST, ALT, cholesterol, and triglyceride. We observed that HDL and cholesterol were highest in G2, with 47.46 and 83.6 mg/dl. The LDL level was highest in G1, at about 17.14 mg/dl. Parameters AST and ALT knew the highest level on G1 with 119.4 and 69.0 mg/dl. On the other side, the highest triglyceride levels were in G5, at about 137.8 mg/dl (Table 2). 

## Discussion

In this study, we induced rats twice a week 0 and 12 by DMPA, with the purpose of suppressing gonadotropin hormones, Follicle Stimulating Hormone (FSH), and Luteinizing Hormone (LH). DMPA inhibits pituitary gonadotropin affecting the endocrine system [18,20]. This condition, in turn, will affect spermatogenesis due to the decrease of the testosterone hormone. We used this model to mimic the male infertility condition and explore improvement with several doses of SE administration. 

In our study, we have shown that SE increased concentration, motility, the viability of spermatozoa, and testosterone levels in DMPA-induced rats. However, the results of all groups showed that the SE treatment group increased the concentration of spermatozoa, and the G3 tended to be higher (52.60 × 10^6^/ml) than the other groups. This is the low dose as the optimal dose to affect spermatogenesis. This result is the same as that reported by Trisnawati [21], that the concentration of sperm was highest in mice. This finding corroborates that Xu et al. [15] stated that the SE from *Hippocampus trimaculatus *Leach and *H. kuda *Bleeker significantly increased spermatozoa in an oligospermic mice model. The content in SE may play a role in spermatogenesis. In another study, we reported the biocompound in SE were alkaloids, steroid glycosides, triterpenoids, and amino acids, with L-Arginine and Glycine as the two highest amino acids [22]. The amino acids are used in Mitogen-Activated Protein Kinase, which increases the expression enzyme Prolyl Endopeptidase in the rat testes, which is regulated by spermatogenesis in mammals [23].

**Table 2.** Serum biochemistry in rats.

Parameters	G1	G2	G3	G4	G5	*p-*value
HDL (mg/dl) Mean ± SD	38.60 ± 6.22	47.50 ± 5.18	44.10 ± 5.08	46.70 ± 32.63	44.40 ± 4.04	0.136
LDL (mg/dl) Mean ± SD	17.10 ± 5.46	11.70 ± 7.01	9.00 ± 3.96	7.10 ± 2.08	9.40 ± 3.85	0.079
AST (mg/dl) Mean ± SD	119.40 ± 16.75	105.20 ± 29.0	98.60 ± 13.50	101.60 ± 34.60	93.60 ± 8.87	0.204
ALT (mg/dl) Mean ± SD	69.00 ± 9.16	62.80 ± 6.61	65.40 ± 6.10	64.60 ± 10.13	64.20 ± 8.37	0.840
Cholesterol (mg/dl) Mean ± SD	73.10 ± 8.35	83.60 ± 10.44	64.70 ± 10.70	71.30 ± 7.75	80.50 ± 14.02	0.115
Triglyceride (mg/dl) Mean ± SD	81.80 ± 13.14	97.80 ± 15.44	98.60 ± 42.61	101.20 ± 20.51	137.80 ± 84.76	0.374

Data with a mean ± SD. G1: Aquadest, G2: CMC 1%, G3: SE dose 150 mg/kg BW, G4: SE dose 225 mg/kg BW, G5: SE dose 300 mg/kg BW.

This study confirmed that motility and viability of spermatozoa were significantly increased in DMPA-induced rats after SE administration. We found that G5 was the highest motility (62.00%) and viability of spermatozoa (64.10%) compared to other groups. This condition is supported by the substance contained in in high doses of SE which is higher than the other groups. Administration of high doses of the extract causes high content of amino acids and steroids. Furthermore, amino acids are used to synthesize Nitric Oxide (NO), which affects the motility and membrane integrity of spermatozoa [24]. In another study, we found the highest amino acids are L-Arginine and Glycine. L-Arginine is a non-essential amino acid which is a precursor of NO [25] and is widely found in seafood and acts as a precursor for the synthesis of putrescine, spermidine, and spermine which affect sperm motility [22]. Indeed, Glycine is one of the homopolymer amino acids as a steroid receptor that affects the androgen receptor involving interactions with Deoxyribonucleic Acid (DNA) and protein that affect the transcriptional activity of androgen receptors [26]. Steroids hormone are cholesterol-derived molecules that regulate various human activities [27], such as the reproductive system in mammals [28]. 

In our study, we demonstrated the beneficial effect of SE on testosterone levels in DMPA-induced rats. Previous studies stated that water extract from seahorse *Hippocampus *sp. significantly increased testosterone levels in mice [21]. Kim et al. [29] also reported that seahorse *H. abdominalis *extract with 25 mg/kg for 12 weeks significantly increased testosterone synthesis in mice *in vivo* and *in vitro*. Our results corroborate the previous studies, which are administrated SE there was an increase with the highest-level tending in G3 (dose of 150 mg/kg BW) around 11.01 ng/dl. This shows that SE content can affect steroidogenesis. One of them is the amino acid L-Arginine is the biological precursor of NO that has significant effects on endocrine function in humans and animals. In rats, NO increases the secretion of growth hormone-releasing hormones and growth hormones. It affects the testis neuronal nitric oxide synthase which affects steroidogenesis by increasing testosterone levels [23].

We observed spermatozoa concentration and testosterone levels decreased with increasing doses. This condition may occur due to an increase in the dose. It can cause testicular damage. If the combination of nutrients is excessive, reductive stress can occur, which increases the concentration of reactive oxygen species. This condition caused DNA damage and reduced the quality of spermatozoa [30]. Oxidative stress is a factor in seminiferous tubule damage [31], which results in cellular changes in steroidogenesis with decreased cAMP production, steroidogenic acute regulatory protein, CYP11A1, CYP17A1, to producing testosterone [32].

Interestingly, our results showed that the increase in sperm motility and viability was not in line with testosterone levels and spermatozoa concentrations. This may be due to dose-dependent, where the dose of the extract was changed, causing a change too in effect. These indicated a possible higher with higher concentrations of the extract, but the minimum dose to induce these effects remained to be elucidated. Besides that, high dose extracts will affect the hypothalamic-pituitary-testis axis in producing Gonadotropin-Releasing Hor through a negative feedback mechanism; then the resulting FSH and LH will decrease. This causes the process of spermatogenesis in the testes to to be disrupted and there is a decrease in spermatozoaand testosterone [33]. 

We observed that SE was relative safety for serum biochemistry, which in all groups showed no significant difference. Regarding HDL levels, we know that the highest level in the group given the SE contrasts with LDL, which is highest in the group without treatment of the SE. This indicates that the endocytosis process through LDL mediation was not optimal in steroidogenesis, decreasing the testosterone and spermatozoa concentrations. For ALT and AST levels, the group with SE tended to be higher than the group without treatment. These facts show that SE does not affect liver function but defends cells from damage. Qian et al. [34] reported that the hydrolysate seahorse could increase the activity of superoxide dismutase and glutathione, which are intracellular antioxidant defense systems. 

Cholesterol levels in our study showed the lowest levels in G3, which aligns with the concentration of spermatozoa and testosterone levels. Cholesterol is a precursor of steroid synthesis in steroidogenesis while increasing testosterone levels [35]. The parameter triglyceride is the highest in G5. We also know that triglycerides consist of fatty acids and glycerol, which have a role as energy from food through conversion in the liver, one of which is in the motility of spermatozoa. However, if the levels are high, it would reduce the concentration of spermatozoa. This fact is in line with our results that G5 is the group with high motility but the lowest concentration of spermatozoa compared to other groups. 

According to our results from the serum biochemistry, this study can be considered safe because they are still within the normal range of rats. These facts can be the based data about the safety level of SE for serum biochemistry profile. We suggested that SE (*Hippocampus comes* L.) as an MNP for sexual prevention drugs in hypogonadism patients and safe for serum biochemistry in rats, indeed a potential candidate from a marine. 

These studies have not comprehensively measured the levels of other hormones which play a role in steroidogenesis and spermatogenesis, including FSH and LH, also the molecular marker in antioxidants. Indeed, further research is needed to ascertain the effect of SE.

## Conclusion

SE (Hippocampus comes L.) dose of 150 mg/kg BW increases sperm concentration and testosterone levels, whereas sperm motility and viability are increased at 300 mg/kg BW in rats induced by DMPA. Nevertheless, SE is a safety for serum biochemistry in DMPA-induced rats. 

## List of Abbreviations

ALT, Alanine aminotransferase; AST, Aspartate aminotransferase; DMPA, Depo medroxyprogesterone acetate; DNA, Deoxyribonucleic acid; ELISA, Enzyme-linked immunosorbent assay; FSH, Follicle stimulating hormone; HDL, High-density lipoprotein; LDL, Low-density lipoprotein; LH, Luteinizing hormone; MNPs, Marine natural products; NO, Nitric oxide; SD, Spraque-Dawley; WHO, World Health Organization.
